# Engineering Cancer Selective Virotherapies: Are the Pieces of the Puzzle Falling into Place?

**DOI:** 10.1089/hum.2022.178

**Published:** 2022-11-14

**Authors:** Emma A. Swift, Steven M. Pollard, Alan L. Parker

**Affiliations:** ^1^ Division of Cancer and Genetics, School of Medicine, Cardiff University, Cardiff, United Kingdom; ^2^ Centre for Regenerative Medicine, Institute for Regeneration and Repair & Cancer Research UK Scotland Centre, University of Edinburgh, Edinburgh, United Kingdom; ^3^ System Immunity University Research Institute, School of Medicine, Cardiff University, Cardiff, United Kingdom.

**Keywords:** gene therapy, cancer, AAV, adenovirus, promoter, enhancer, oncolytics, immunotherapy, capsid engineering, rational design, directed evolution, therapeutic payloads

## Abstract

Advances in gene therapy, synthetic biology, cancer genomics, and patient-derived cancer models have expanded the repertoire of strategies for targeting human cancers using viral vectors. Novel capsids, synthetic promoters, and therapeutic payloads are being developed and assessed through approaches such as rational design, pooled library screening, and directed evolution. Ultimately, the goal is to generate precision-engineered viruses that target different facets of tumor cell biology, without compromising normal tissue and organ function. In this study, we briefly review the opportunities for engineering cancer selectivity into viral vectors at both the cell extrinsic and intrinsic level. Such stringently tumor-targeted vectors can subsequently act as platforms for the delivery of potent therapeutic transgenes, including the exciting prospect of immunotherapeutic payloads. These have the potential to eradicate nontransduced cells through stimulation of systemic anticancer immune responses, thereby side-stepping the inherent challenge of achieving gene delivery to the entire cancer cell population. We discuss the importance of using advanced primary human cellular models, such as patient-derived cultures and organoids, to enable rapid screening and triage of novel candidates using disease-relevant models. We believe this combination of improved delivery and selectivity, through novel capsids and promoters, coupled with more potent choices for the combinations of immunotherapy-based payloads seems capable of finally delivering innovative new gene therapies for oncology. Many pieces of the puzzle of how to build a virus capable of targeting human cancers appear to be falling into place.

## INTRODUCTION

Despite an array of improved surgical, chemotherapeutic, and radiotherapy interventions, options for cancer treatment remain inadequate to meet the needs of all patients. While new molecularly targeted therapies and immunotherapies have been successful in controlling certain tumor types, complete and durable remissions are often restricted to a disappointingly small subset of patients. Hence, cancer remains a leading global cause of death. There is an urgent need to establish innovative novel therapeutic strategies that are effective against tumors with the poorest survival rates. As a fundamentally genetic disorder, developing gene-based therapies for cancer has long been a seductive notion.

Thus far, the greatest accomplishments in the gene therapy field have come from the replacement or correction of single mutated genes to restore normal function in monogenic disorders. If the resulting protein operates through paracrine or systemic mechanisms, expression from only a subset of cells is needed to achieve meaningful clinical impact (*e.g*., a missing enzymatic activity). This can easily be achieved by means of viral-mediated gene transfer, including by vectors based on adeno-associated viruses (AAVs) and adenoviruses. These have been effective across a range of monogenic disorders with licensed therapies approved for treatment of monogenic disorders, including inherited retinal dystrophy (Luxturna™), spinal muscular atrophy (Zolgensma™), and hemophilia (Roctavian™). Nevertheless, the deaths of four patients in the recent Astellas AAV-based gene therapy clinical trial for X linked myotubular myopathy (XLMTM)^1^ serve as an unfortunate reminder that improvements in viral selectively are required for dosing within appropriate therapeutic windows.

Adopting a similar strategy to correct cancers by delivering wild-type tumor suppressor genes to tumors using a gene therapy approach has been the focus of a great deal of research. In 2003, a replication-deficient adenovirus expressing wild-type p53, Gendicine™, was approved in China for the treatment of cancer (reviewed in Zhang et al^2^). Despite the appeal of this strategy, adopting a similar approach to deliver functional tumor suppressor genes or correct oncogenic driver mutations in cancer is plagued by two perennial issues: first, the heterogeneity of genetic disruptions in most aggressive solid tumors leads to plasticity and redundancy in oncogenic signaling pathways.

Hence, correction of no single key mutation would suffice (in most circumstances) to restore normal cellular behavior. Second is the delivery challenge, where prevention of tumor regrowth would only be accomplished if 100% of tumor cells we corrected. This is an impossible feat for even the most efficient methods of gene transfer. As such, despite decades of interest and exploration, development of gene-based therapies for cancer has lagged significantly behind those for monogenic disorders.

Nevertheless, there is growing excitement and optimism that gene therapies have matured sufficiently to be harnessed in oncology. Success will most likely require the delivery of therapeutic transgenes that target different facets of tumor cell biology, rather than correction of underlying genetic driver mutations. Tumor-localized expression of therapeutic payloads that re-invigorate the anticancer immune response, inhibit angiogenesis, target the immunosuppressive tumor stroma, or convert prodrugs into their active cytotoxic form can target universal biological vulnerabilities of cancer cells. The remaining challenge is how best to deliver such potent payloads to tumor cells, while minimally impacting healthy tissue.

## THE POTENTIAL OF VIRAL-BASED GENE THERAPY TO TREAT HUMAN CANCERS

Viruses are naturally pathogenic agents responsible for a broad spectrum of diseases and associated clinicopathological symptoms. Yet when harnessed appropriately, they enable highly efficient targeted delivery of nucleic acids, and hence have been the favored choice for the delivery of exogenous DNA transgenes to human tissues. The relative ease of genetic manipulation and capacity to be produced to high titers and degrees of purity further add to excitement regarding their potential application in cancer gene therapy.

The range of oncolytic and viral gene therapy vectors being developed in the cancer field is diverse, encompassing both RNA and DNA viruses. In this review, we focus our discussion on examples from adenoviruses and AAV-based vectors. However, the strategies and challenges outlined are broadly applicable to other viral vectors.

Adenoviruses drive robust, transient, high-level expression of transgenes, making them ideally suited to cancer gene therapy applications. Furthermore, recently, there has been renewed interest in oncolytic virotherapy, which utilizes replication-competent vectors such as conditionally replicating adenoviruses. A partially intact E1-gene enables intratumoral viral replication, resulting in direct oncolysis of transduced tumor cells, as well as *in situ* amplification of the therapeutic agent.^3^ Viral progeny can spread through the tumor, causing further destruction of malignant tissue and release of tumor antigens. This results in a multipronged attack on tumors that can synergize with other immunotherapeutic cancer targeting strategies and offers a clear advantage over nonviral means of gene transfer.

By contrast, AAVs are ideally suited to long-term gene corrections strategies and form the platform of an ever-expanding number of licensed gene therapy products. In relation to cancer, AAVs offer the prospect of more durable gene expression that might be required for certain tumors to eliminate residual quiescent and dormant cells, since AAVs can transduce both dividing and nondividing cells and the viral genome persists as an episome. Use of AAVs has therefore been explored in both preclinical and clinical studies using a range of payloads (reviewed in Santiago-Ortiz et al^4^). The physical size (∼25 nm) is smaller than adenovirus and may facilitate better biodistribution in certain tissues and there is evidence of good transduction from the natural serotypes across a range of cancer types, most likely due to the roles of heparan sulfate, integrins, proteoglycans, and RTK as receptors/co-receptors—expression of which is elevated in many solid tumors.

However, a potential limitation of AAV application in oncology is rapid loss from dividing cancer cells—and so the proliferative index of the tumor type of interest might dictate the best choice of vector. For example, in glioblastomas, a significant proportion of the tumor is noncycling and quiescent/dormant, posing a challenge to transient adenovirus,^5^ while AAV would likely persist longer, facilitating clearance of residual disease after chemotherapy/radiotherapy. The widespread delivery and persistence of AAV can of course pose risks if the anticancer payloads are detrimental to normal tissue; hence, ensuring limited off-target expression or transduction of normal surrounding tissues is especially important for AAV compared to adenovirus.

A prerequisite for any successful cancer therapy is the ability to target cancer cells selectively, while sparing the body from local or systemic toxicities arising from off-tumor effects.

**Figure 1.**
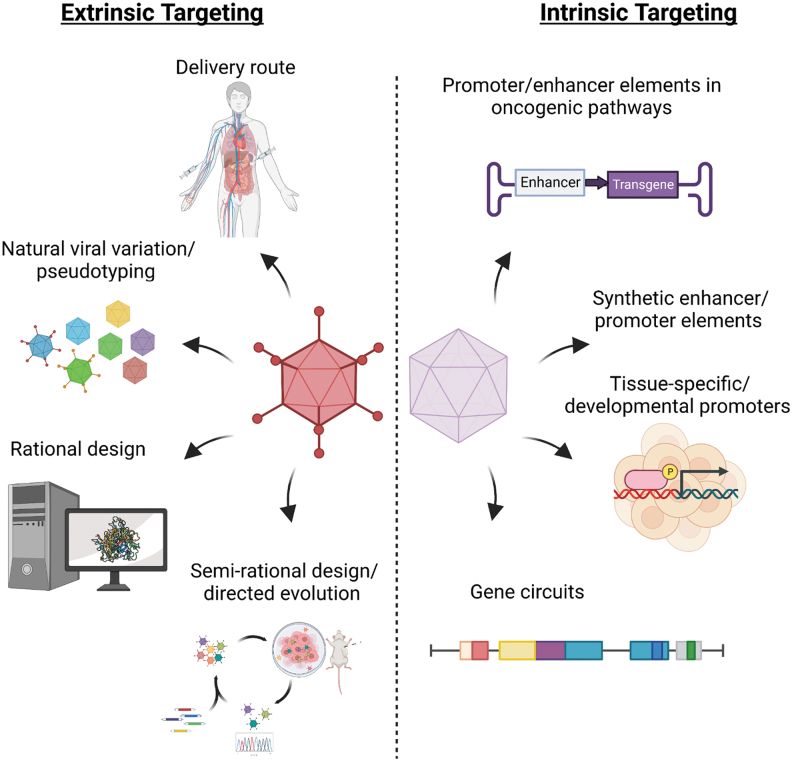
Summary of extrinsic and intrinsic strategies for engineering cancer-selective virotherapies. Extrinsic methods may encompass combinations of route of delivery, pseudotyping approaches, as well as knowledge-guided rational or semirational vector engineering approaches. Intrinsic selectivity may be achieved through combinations of tissue- or tumor-specific enhancer or promoter sequences, gene circuits, or alterations in early viral genes that enable selective viral replication within tumor cells.

Below, we discuss progress and challenges in the approaches that can address this urgent need to restrict the activity of virotherapies to the malignant cell compartment. These encompass both cell extrinsic and intrinsic engineering opportunities (overviewed in Fig. 1). We focus on how optimization of delivery route, capsid design, and promoter/enhancer regulation of viral gene expression can generate precision targeted vectors for cancer virotherapy. These can subsequently be armed with potent therapeutic payloads to ensure maximum efficacy with minimal off-target damage.

## CELL EXTRINSIC OPTIONS FOR GAINING SELECTIVITY

Extrinsic targeting strategies enable selectivity to be imposed before the virus has even entered the cell. The aim is to generate genuinely tumor-tropic viruses that exclusively infect tumor cells, thus avoiding sequestration in normal tissues. This reduces the required dose and risk of tissue toxicity, while simultaneously increasing the amount of payload that “hits” the tumor. Directing viral vectors to tumor cells has been explored by several approaches, including optimizing the route of delivery, choice of virus, and altering the array of cell entry receptors through capsid protein modifications (Fig. 1).

### Route of delivery: local or systemic?

Some level of control can be achieved through astute selection of the route of delivery of the virotherapy into the patient. The route of administration has a direct bearing on the range and order of tissues that will be encountered and is therefore an important determinant of vector tropism. Systemic delivery, through the bloodstream following intravascular administration, necessitates trafficking of the vector to the tumor. Local delivery encompasses both direct intratumoral delivery and various site-specific injections that provide the vector near lesions at different anatomical locales (*e.g*., intraperitoneal injections for tumors localized within the abdominal cavity, intrathecal injection for tumors of the central nervous system [CNS]).

Intratumoral injections are presently the most commonly relied upon approach for administering virotherapies to patients.^6^ This is unsurprising, given the stringent control over the exact dose reaching the tumor and more favorable safety profile resulting from restricting delivery to the site of need. Local delivery further avoids exposure to blood plasma proteins, including pre-existing neutralizing antibodies.^7^ For some viral serotypes, such as the heavily relied upon human adenovirus serotype 5 (Ad5), seroprevalence rates in specific geographical cohorts can be extremely high (up to 80–90%),^8^ potentially limiting the widespread effectiveness of systemically delivered therapeutics based on these viral backbones in specific populations. Other plasma factors can also be problematic; the clotting factor FX, for example, can promote rapid and efficient liver sequestration of intravenously delivered Ad5-based therapeutics through cross-linking to heparan sulfate proteoglycans (HSPGs) on hepatocytes.^9,10^

Despite the advantages of local delivery, the ability to effectively administer virotherapies through intravenous injection remains a long-standing goal. Systemic delivery of virotherapies affords the opportunity of transducing the greatest number of tumor cells as intravenously injected viral vectors will travel through the bloodstream and can therefore potentially engage with all tumor cell populations, including disseminated metastatic or micro-metastatic lesions, which may not be detectable by conventional imaging methods. At a practical level, intravenous injection is facile, subverting the need for the complex surgical procedures that are required to access difficult-to-reach tumors for direct injection.

Furthermore, easily accessible tumors in noncritical organs are unlikely to benefit from intratumorally delivered virotherapies, since these are more amenable to conventional surgical resection—the preferred therapeutic stratagem. However, treating local tumors with oncolytic virotherapies may initiate immune priming and systemic abscopal effects.^11^ Overcoming the systemic delivery challenge and identifying viral vectors that preferentially transduce tumor cells are therefore of the utmost importance; this will make trials easier and safer, as well as increasing the opportunity to eradicate all tumor cells.

There have been a few studies investigating the clinical efficacy of intravenously delivered viral cancer therapy vectors.^12^ Those that have completed typically failed to demonstrate clear improvements in primary endpoints, likely reflecting the significant challenges posed by immune-clearance and off-target sequestration. Nevertheless, these challenges are not insurmountable, and improvements may be made through modification of the vector capsid surface to evade immune recognition and increase the selectivity over target-cell transduction. With the tools of modern synthetic biology, it has been possible to develop strategies that facilitate the development of viruses with genetically and chemically modified capsid proteins. Successes in rodent models, discussed below, point to the potential for highly tumor-selective viruses to be engineered for human applications.

### Capsid engineering: capitalizing upon natural viral variation

Human adenoviruses comprise a diverse phylogenetic tree containing 57 canonical serotypes. This diversification arises from natural selection pressures, resulting in viruses that differ in their tropism, cytotoxic capacity, and cellular infection kinetics.^13^ Adenoviruses are classically divided across seven different human adenoviral species from A to G; these engage diverse cell entry receptors, ranging from the tight junction localized coxsackie and adenovirus receptor (CAR) for species A, C, E, F, and (to a lesser extent) D, to CD46 and desmoglein-2 (DSG-2) for species B viruses, and sialic acid for certain members of species D.^14,15^ This variation can be capitalized upon for improved vector design by enabling the selection of serotypes that possess more favorable properties in the context of anticancer therapeutics.

Despite its prevalent use as a gene therapy vector, the species C adenovirus type 5 (HAdV-C5) has a broad tropism resulting from its reliance on CAR as a primary entry receptor, expression of which has further been shown to be downregulated on numerous cancer subtypes. The ability of the Ad5 hexon to bind FX protein and traffic to the liver, coupled with high seroprevalence rates in certain populations, make the investigation of alternative serotypes with lower seroprevalence rates and unstudied tropisms worthwhile for virotherapy applications. In this regard, species D adenoviruses (the largest human adenoviral species) represent a relatively untapped pool of potential backbones with far lower seroprevalence rates,^10,16^ which do not bind FX in the blood.^10^

The species D adenovirus serotype 10 (HAdV-D10, Ad10) has recently been shown to form weak interactions with known adenoviral receptors, therefore providing a compelling starting point for engineering more cell type-selective infection and lysis of cells (*in vitro* and *in vivo*).^17^ HAdV-D 26, 28, 45, and 48 have been shown to infect and replicate in several cancer cell lines, although cytotoxicity was lower than adenovirus 5-based vectors.^18^ Moreover, a replication-competent species B Ad11-derived vector was demonstrated to be capable of infecting and inducing oncolysis in colon cancer cell lines, which expressed high levels of the CD46 receptor.^19^ These studies highlight the feasibility of generating virotherapies from noncanonical human adenoviral serotypes.

Efficient cellular transduction, potent cytotoxicity, and wealth of knowledge surrounding the use of Ad5 remain attractive features of this vector; hence, it remains a leading contender in cancer virotherapy development. To take advantage of these desirable traits, while simultaneously adjusting the cellular tropism to one more suitable for cancer targeting, a heavily adopted approach has been to generate pseudotyped viral vectors. This involves the replacement of either the C-terminal fiber knob domain or the entire fiber protein with that from another adenoviral serotype.^20^

This radically changes the receptors that can be engaged for cellular transduction.^21^ For example, pseudotyping the fiber knob of adenovirus 5 for that of species B adenovirus 3 (Ad3) alters primary receptor usage to render cellular infection CAR independent, with the resulting Ad5/3 pseudotype virus displaying enhanced transduction and oncolysis of ovarian carcinoma cell lines *in vitro* and in immunocompromised murine xenograft models^22,23^; this was due to cell entry through DSG-2, the primary receptor for the Ad3 virus.^24^

Pseudotyping the CD46-binding Ad35 fiber has also proved particularly promising, enabling enhanced gene delivery to human smooth muscle, CD34^+^ hematopoietic cells, gliomas, and various other tumor cell lines.^21,25–27^ The length of the adenovirus fiber has been shown to be important in impacting interaction with cell surface receptors and subsequent αv-integrin-mediated virus internalization.^28^ Care must therefore be taken to consider the impact of any alteration in fiber length when exchanging the entirety of this capsid protein. Nevertheless, the successful generation of several Ad5 fiber pseudotyped viruses demonstrates the feasibility of this approach.

For AAV, there are around 13 different serotypes that are widely available, each with distinct capsid proteins that underpin their distinct tropisms. Natural serotypes have strikingly different cell type selectivity, for example, when comparing these in parallel in the primate CNS.^29^ The reader is directed to Samulski and Muzyczka, for a comprehensive review of AAV biology, the history of its discovery, and its importance in gene therapy.^30^ It is relatively straightforward to engineer altered capsids (encoded by the polycistronic viral *cap* gene) and produce recombinant AAV (rAAV) that can be tested with distinct genes of interest flanked by the common AAV-2 inverted terminal repeats (ITRs). The AAV capsid is responsible for the binding to various proteoglycans and both primary and secondary receptors. With improved knowledge of the key biochemical structural features required for receptor interactions, opportunities have opened for rational capsid engineering, often focusing on a specific region of the VP2 loop.^31^

### Capsid modifications

**Figure 2.**
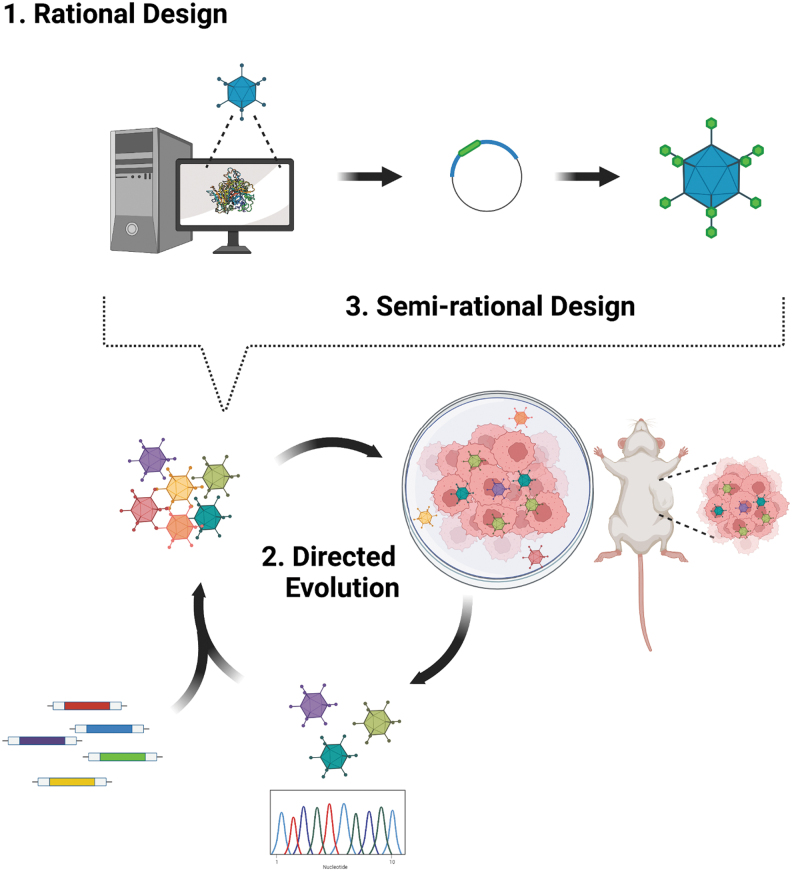
Strategies for the development of genetically re-targeted cancer virotherapy vectors. Knowledge of capsid protein structure can be harnessed to aid in the rational design of virotherapy vectors with novel binding interactions **(1)**. Alternatively, a structure-free directed evolution approach may be employed, in which initial diverse pools of potential viral vectors are subjected to selection on cell populations of interest, either *in vivo* or *in vitro*. Virions recovered from this can be sequenced and further diversified (*e.g*., by error-prone PCR) followed by use as the input for subsequent rounds of selection. After multiple rounds, viruses with improved replication and infection kinetics in the cell line of interest can be isolated **(2)**. These two approaches can also be combined in a powerful semirational design approach. Here, structural knowledge can guide the insertion of random targeting molecule libraries into permissive regions of the viral capsid. Insertions that improve cancer-selective targeting can be selected for by subjecting the resulting viral pool to high-throughput directed evolution screening **(3)**. PCR, polymerase chain reaction.

A major limitation of relying on the natural viral variation to achieve cell extrinsic selectivity is that the array of receptors available is restricted to those naturally encountered and selected during viral evolution. Rational design may be a way to expand viral tropism usefully to enable targeting of transformed cells. As such, the extensive body of knowledge surrounding viral capsid structure and interaction with host receptors can be leveraged to generate precise tropism-modifying alterations that improve the tumor selectivity of vectors (overviewed in Fig. 2). This approach has great potential to yield highly selective novel anticancer virotherapies; not only can it aid in the identification of mutations that ablate interactions with broadly expressed native receptors but can also reveal permissive sites for the insertion of new targeting moieties that redirect viral tropism toward cancer cell populations of interest.

An example of this is the insertion of peptides that support binding to extracellular matrix (ECM) or their receptors that are enriched in solid tumors, such as tenascin, LeX, or integrins.^32^ This combination of viral de- and re-targeting is a prerequisite for the development of precision-targeted virotherapies that traffic exclusively to tumor cells, especially following systemic administration.

Attempts have been made to nongenetically alter viral tropism such as through chemical coupling of capsid proteins to polymers, such as polyethylene glycol (PEG),^33^ and poly-[N-(2-hydroxypropyl)methacrylamide] (pHPMA).^34^ These decorate the viral surface in a way that abrogates interaction with native receptors as well as plasma proteins, potentially enabling evasion of immune-mediated clearance by pre-existing antiviral antibodies. Targeting toward specific cellular subpopulations is subsequently achievable through coupling of the capsid to multivalent polymers, antibodies, peptides, or other high-affinity moieties that bind the receptor of interest.^35,36^

Other nongenetic targeting strategies utilize bi-specific adaptors such as Fab-based fragments, scFv diabodies,^37^ bi-specific DARPins,^38^ and single-chain diabodies to simultaneously engage capsid proteins and cancer-associated receptors through opposite poles of the molecule.^39^ These nongenetic strategies have the limitation of an increased complexity associated with the manufacturing, quality controls, and delivery of such multicomponent systems. Furthermore, this retargeting strategy lacks heritability of tropism, which is of concern for replication-competent viral vectors where nontargeted daughter virions produced *in situ* could mediate off-target effects. As such, genetic modification of adenoviral tropism is a more desirable strategy.

Noteworthy for adenovirus has been the application of rational design to generate genetically targeted vectors that lack affinity for the ubiquitously expressed CAR receptor.^40^ Binding can be abolished through two amino acid substitutions (S408E, P409A; referred to as KO1 mutation) in the Ad5 fiber knob AB loop,^41^ or a Y477A substitution and TAYT deletion in the FG loop.^42^ These have been widely incorporated into cancer-targeted adenoviral-based therapy vectors to prevent CAR-dependent cellular transduction in off-target healthy bystander tissues. Despite these tropism alterations, Alemany and Curiel showed that liver sequestration remains an issue.^43^

Further modifications within the remaining two major capsid proteins—the hexon and penton—eliminate the residual interactions with FX and αvβ3/5 integrins, respectively.^44^ These encompassed modification of the hexon hypervariable region 7 and an RGD to RGE mutation in the penton base. The resulting vector, Ad5_NULL_, is totally devoid of native means of uptake. Such vectors provide invaluable “blank canvases” on which further re-targeting modifications may be superimposed to generate precision-targeted virotherapies that are exquisitely tuned to cancer cell transduction.

The site of insertion of targeting moieties must be carefully considered to avoid disruption of viral packaging and nuclear trafficking, while facilitating presentation to tumor-associated receptors of interest. Several capsid locales have been evaluated, including the hypervariable loops of the hexon,^45,46^ fiber,^47^ and minor structural protein pIX of the adenovirus.^48^ Whilst the high copy number of the hexon and pIX proteins (720 and 240 copies per virion, respectively) makes these ostensibly appealing sites, conferring novel high affinity receptor interactions here may compromise the downstream virus release from endosomes following internalization.

By far, the most common site for rational insertion of targeting scaffolds has been the knob domain of the adenovirus fiber.^47^ The surface-exposed HI loop of the HAdV-C5 fiber knob has been shown to be particularly tolerant of heterologous insertions, with peptides in excess of 100 amino acids being incorporated without detriment to viral fitness.^49,50^ Homology modeling has revealed the DG loop of the Species D adenoviruses 10 and 48 to be similarly permissive to insert engineering.^17,51,52^ The C-terminus of the HAdV-C5 knob domain has also previously been shown to enable virus re-targeting without compromising capsid assembly,^53^ while another approach has been to remove the fiber knob entirely and replace this with domains that facilitate incorporation of larger targeting molecules without structural clashing such as the T4 bacteriophage-derived fibritin protein.^54^

An extensive range of scaffold proteins with variable affinities have been rationally engineered into the adenoviral capsid structure to facilitate retargeting. These range from peptides, and scFvs, to T-cell receptors (TCRs), DARPins, and nanobodies. Of note are RGD motif-containing peptides that facilitate viral retargeting to integrins. Integrins are frequently overexpressed on aggressive carcinomas, while displaying relatively limited presence on healthy human tissues. This interaction could therefore be harnessed to achieve highly specific cancer targeting. Incorporation of the FMDV2 derived, αvβ6 integrin targeted A20 peptide into the HI loop of the fiber knob of the Ad5_NULL_ platform has been shown to facilitate tumor-restricted transduction and transgene expression in an immunocompromised mouse xenograft model.^44,55^ Similarly, DARPins for cancer-overexpressed receptors, such as HER2/neu, have also been demonstrated to facilitate cancer-specific delivery for AAV.^56^

## UNBIASED SCREENING WITH DIRECTED EVOLUTION

A complementary approach to rational engineering discussed above is to use directed evolution strategies that do not require any *a priori* knowledge of the underlying receptor-ligand interaction—although often do focus on specific structural regions. Highly diverse initial libraries are subjected to iterative rounds of *in vitro* or *in vivo* screening for transduction of cancer cells of interest with normal cell controls, to select for those virions that have improved transduction profiles and/or infection kinetics (Fig. 2).

The starting viral pool may be composed of a mixture of naturally occurring viral serotypes, which are encouraged to undergo recombination during serial passaging, producing chimeric viruses optimized for target cell killing. Proof of principle of this was demonstrated by Kuhn et al in a study in which culture of a mixture of adenoviral serotypes from across species B-F with the colon cancer cell line HT-29 led to isolation of an Ad3/11p chimeric virus termed ColoAd1.^57^ This was demonstrated to have increased lytic capacity and selectivity for transduction of colon cancers when compared to the parental serotypes.

These approaches, conducted in cell culture *in vitro*, tend select for subtle advantageous recombinants, with alterations in early proteins enhancing the intrinsic properties—that is, for alterations that enhance viral replication in and killing of tumor cells, rather than extrinsic tumor selectivity *per se*. Potentially, the most promising approach may combine rational design with the high-throughput screening afforded by directed evolution.

Inserting large libraries of diverse protein or peptide domains (*e.g.*, DARPins or scFvs) into identified permissive capsid sites enables iterative rounds of screening and recovery of semi-rationally designed vectors, with mutagenesis by synthesis of random variants providing further diversification. By starting with a pool of initial viruses whose design was informed by pre-existing knowledge of structural biology and using protein sequences with anticipated activity, the likelihood of identifying a lead candidate vector for the desired purpose at the end of this process is greatly increased. This semirational approach has been adopted within the AAV field.^58^ However, increased complexities surrounding the generation of in-context adenoviral display libraries mean this approach is yet to produce significant breakthroughs for these vectors.^59^

For AAV, an example of the power of screening random peptides to yield capsids with improved binding properties has been achieved in the nervous system, reviewed by Challis et al.^60^ Using elegant genetic screening in mice, Deverman et al were able to identify the PHPeB capsids, which displayed remarkable tropism for the adult CNS, crossing the blood–brain barrier,^61,62^ although these have not proven effective in primate studies, due to mouse strain-specific molecular mechanism (SCA-1 binding) underlying the activity.^63^ These studies nonetheless show the power of using sophisticated genetic screening strategies to recover desirable capsids.

Other innovative approaches for delivery, such as exosomes, nanoparticles, or synthetic cells, may emerge in coming decades. However, at present, these are unlikely to supersede engineered viruses. Discussion of these nonviral methods for delivery is beyond scope of this review. For the moment, evolution has resulted in very efficient gene delivery vehicles for human cells in the form of viruses; tapping into their vast potential for engineering selectivity remains a priority for the field.

## CELL INTRINSIC OPTIONS FOR GAINING SELECTIVITY: TUMOR-SELECTIVE REPLICATION AND CELL TYPE-SELECTIVE PROMOTERS

Following internalization, additional control can be exerted by regulating expression of payloads and/or critical viral replicative genes (for adenovirus) using promoters and enhancers that restrict their transcription to the malignant cellular compartment. Arguably, the selectivity that can be achieved through engineering of promoter/enhancers may far outstrip that which could ever be achieved through capsid engineering, since capsids are likely constrained by limitations inherent in the biology and biochemistry of available interactions. By contrast, modular regulatory elements and “tuning” of expression for specific cell types and states can be spectacularly selective as there is a combinatorial logic in the transcription factor (TF) “code,” which underpins the identity of many thousands of diverse cell types and states that make up human tissues.

Historically, ubiquitous viral promoters have been favored for cancer gene therapies. These provide constitutive, high-level expression of the transgene. However, there are two broad choices that might give desirable selectivity by ensuring transcription in only tumor cells: (1) use of a tissue/developmental specific elements that are reactivated or highjacked in the tumor cells, but not normally present in adult tissues (*e.g*., alphafetal protein for hepatocellular carcinoma^64^ or carcinoembryonic antigen [CEA] for colon cancer^65^) and (2) promoters/enhancers that are the endpoints in classic oncogenic signaling pathways (*e.g*., E2F) (reviewed in Nettelbeck et al^66^), cancer-specific mutations (*e.g*., telomerase, TERT promoter^67^), or hypoxic elements.^68^ Other examples are oncogenic pathways, including the use of the oncogene-associated promoters. These genes and hence their associated promoters would display higher activity in cancer cells than surrounding tissues, providing therapeutic window.

These early studies focused on proximal promoter regions; yet, for many cell type-specific genes, it is the long range cis-regulatory elements (enhancers) that provide the opportunity for selectivity.^69^ Since ∼2000, there has been an explosion of technologies and methods that now enable rapid identification of candidate enhancers (cell type specific) through mapping of peaks for key TFs using ChIP-seq, enhancer chromatin markers such as H3K27 acetylation, or chromatin accessibility (ATAC-seq). This has resulted in thousands, or tens of thousands, of candidate cell type-specific regulatory elements that might be useful in adenoviral or AAV to provide selective expression.

Moreover, advances in single-cell RNA-seq, DNA methylation, and chromatin accessibility profiling mean we will soon have a comprehensive atlas of diverse human cell types and cell states, with maps of their associated enhancers, including for tumor cells. Functional annotation of these regulatory elements alongside computational tools and machine learning strategies to identify the “rules” underlying their activity should transform our ability to rapid garner optimal synthetic promoters for gene therapy.

As we learn more about the key TFs that define cell type identity, their specific motifs, and the grammar that leads to their selectivity,^70^ these can be readily synthesized and screened (as they are often short sequences) for specific activity. Quicker, cheaper, and scalable methods to read, write, synthesize, and assemble DNA underpin the relatively new field of synthetic biology (*e.g*., automation, golden gate, Gibson assembly, massively parallel DNA synthesis). Novel synthetic promoter and enhancers are therefore increasingly very easy to design, build, and test.

Screening approaches for synthetic promoters and enhancers follow a similar strategy to capsids and involve similar steps: building complex libraries of DNA parts, designing strategies to identify the “winners,” and recovery of those regulatory sequences with desired activity using appropriate human cells or animal models. An example of what is possible is the screening in mice for 230 different enhancer sequences in AAV, which uncovered many novel elements that are specific for neuronal and glial cell subtypes. Furthermore, Mich et al demonstrated an elegant *in vivo* screening strategy searching for neuronal subtype enhancers revealed by scATAC-seq and identified those that can operate in AAV across species.^71,72^ Single-cell profiling technologies therefore not only provide the input set of candidate enhancers but also can be used to functionally annotate and rank these within their appropriate rAAV context.^73^

Natural enhancers may have the wrong size, selectivity, or strength to be useful in gene therapy applications. For fully synthetic enhancers, most efforts initially have focused on transcription factor binding motifs (TFBMs; 5–10 bp) and concatemerization of these to gain stronger activity.^74^ This approach has been used to search for glioblastoma promoters using screening of libraries.^75^ However, this assumes that the key TFBMs are known and ignores the need for the appropriate “grammar” that will undermine either activity (strength) or selectivity. Future trends will likely combine the use of natural elements screened at scale that can be minimized (to retain the key natural grammar of motifs), but combined in ways to rationally design cell state or type specific expression.

## CANCER-SELECTIVE PAYLOADS

**Figure 3.**
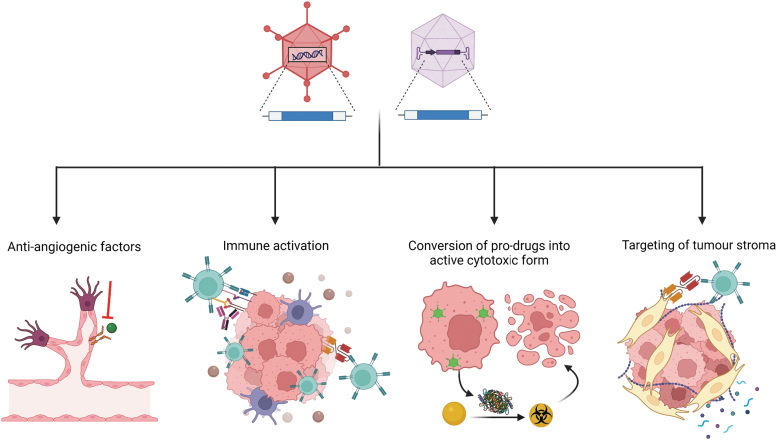
Arming cancer virotherapies with therapeutic payloads. Cancer virotherapy vectors can mediate efficient delivery of therapeutic transgenes that target multiple different aspects of tumor cell biology. This includes factors that inhibit angiogenesis, re-invigorate the anticancer immune response, promote tumor cell lysis through localized activation of cytotoxic drugs, and target the dense immunosuppressive stroma.

Whilst replication-competent adenoviral-based therapies have some intrinsic anticancer activity through their oncolytic capacity, potency can be greatly potentiated by the incorporation of additional transgenes that confer novel therapeutic functionalities—effectively enabling them to act as Trojan horses that deliver anticancer payloads to the tumor cells. An array of different transgenes has been explored within the context of cancer virotherapy vectors (overviewed in Fig. 3), these are united by the fact that their anticancer effects can extend beyond the cell from which they are expressed to impact upon neighboring untransduced cells.

This “bystander effect” is critical for ensuring clearance of the entire tumor rather as opposed to exclusively the transduced cell subset. The ability to induce an immunological memory response is further desirable since this may enable protection against recurrence. How close are we to achieving this? Certainly, great steps toward achieving this “holy grail” have been enabled by the emergence of immunotherapeutic payloads; with the successes of checkpoint inhibitors for melanomas and CAR-T approaches for leukemia, the immunotherapy field has blossomed and is stimulating new discoveries and likely a wealth of candidate payloads that might be suitable for local viral mediated delivery.

### Arming with immune-stimulating anticancer transgenes

Many solid tumors develop, by immunoediting, to acquire mechanisms that are immunosuppressive—most obviously, through downregulation or deletion of MHC or associated antigen-presenting apparatus, as well as evolving a myelosuppressive microenvironment with increasing concentrations of immunosuppressive immune cells such as regulatory T cells (Tregs), tumor-associated M2 polarized macrophage, and likely multiple other pathways. Hence, educating or unleashing the immune system through local tumor-specific immune modulators is an appealing strategy. The successes of immunotherapy for certain cancers and massive interest and investment in this area mean there is an ever-expanding collection of potential immuno-therapeutic payloads with which to arm virotherapies.^76^

Encoding such immune modulators within the framework of a cancer-selective virus can help to bypass some of the issues associated with their systemic application such as immune system hyperactivation, which in the worst cases can lead to systemic cytokine storms and ultimately death. Restricting the activated immune response to the tumor and its local microenvironment is one of the appealing prospects for viral gene therapies.

Proof of principle of the ability of immune transgenes to provide therapeutic efficacy when encoded as part of cancer virotherapies comes from the first oncolytic virus to be approved in the United States and Europe in the form of Talimogene laherparepvec (Imlygic™). This is an HSV-1-based virotherapy that incorporates a transgene encoding granulocyte macrophage colony-stimulating factor to promote the proliferation and differentiation of the bone marrow myeloid precursor cells that give rise to important immune effector subsets. Additional examples of immune-activating transgenes that have been encoded within the viral genome include checkpoint inhibitors, cytokines, chemokines, and bi- or tri-specific NK and T cell engagers.^3,76^

Typically, the latter of these possess two scFv domains that enable them to engage with tumor-associated antigens at one end and the relevant immune cell subset at the other, thus bringing the two into proximity for immune cell activation and subsequent tumor clearance. IL-12 has previously been explored as a potential antitumor cytokine, with extremely promising preclinical studies that were subsequently undermined by toxicities when systemically delivered in clinical trials.^77^ However, the use of IL-12 in a more restricted manner with viral delivery has shown some signs of success in glioblastoma.^78^

Finally, viruses with oncolytic capacity, such as adenovirus, have the potential to provide particularly powerful synergies with immune-stimulating anticancer transgenes, since the immunogenic cell death that follows vector self-amplification and subsequent cell lysis releases damage-associated molecular patterns and tumor cell antigens that lead to recruitment and activation of immune effector cells, which potentiate the effects of any transgene.^79^ Oncolytic activity in adenoviral constructs is commonly conferred by subtle changes in viral early genes that enable replication to proceed in tumor cells, yet attenuated in nontransformed cells. One such modification is the deletion of the adenoviral E1B protein, and this modification is the basis of Oncorine™, which has been licensed in China since 2005 for the treatment of nasopharyngeal carcinoma and head and neck cancers (reviewed in Wei et al^80^).

## THE IMPORTANCE OF PATIENT-DERIVED TUMOR MODELS

Having designed precision-engineered viral vectors and armed them with therapeutic payloads for efficacy, screening and testing in appropriate models must be performed to identify the most promising candidates for clinical translation. A key concern of any screen or cell line/rodent-based studies for a human therapeutic agent is that the experimental model will reveal highly selective promoters or capsids that only operate in that specific model due to its unusual biology—and may have limited utility across species. The advent of primary human cancer models provides a timely experimental advance that can help address these limitations.^81^ These patient-derived cancer models can provide improved *in vivo* models when orthotopically transplanted into mouse tissues (immunocompromised mice). These xenografts have more realistic histology and virus activity can therefore be explored through different delivery routes.

Patient-derived monolayers or organoids enable regulatory elements and viral vectors to be tested in the most relevant human disease context. Three-dimensional organoids that have the heterogeneity, hypoxic microenvironments, and diversity of differentiated states seen in primary tumors are a more realistic model for screening and head-to-head comparisons can be made with normal nontransformed tissue control organoids.^82^ Solid tumors, including patient-derived models, are now widely available and often are shared to the community and industry.^83^

There are of course caveats. Patient-derived cultures lack vasculature and immune cells; they are often not easy to scale up and engineer genetically. Furthermore, they may lack other features of the tumor biology such as appropriate extracellular matrices or structural features/mechanical forces. With long-term passage, there is the risk of genetic drift. However, as a complementary approach to murine studies, human primary cell cultures, organoids, and slice cultures should always be part of the triage process for identifying optimal engineered viruses for gene therapy.

This ability to move back and forth between *in vitro* and *in vivo*, with normal matched tissue stem cell controls, should be harnessed in all discovery research to optimize and triage candidate lead products before entering the clinic. A major limitation, however, is the difficulty of modeling human immune interactions with the organoid-derived tumors. While strategies have been developed, these are often technically challenging, artificial, and costly; co-cultures of organoids with vascular cells and/or human immune cells may be more tractable.

## CONCLUDING REMARKS

In conclusion, viral-mediated therapeutic gene delivery could achieve cancer-selective killing or cytostatic effects if the virus preferentially transduced cancer cells, only replicated within the cancer cells, or delivers payloads that are only expressed or active within the cancer cells (or a combination of these features). Over the past two decades, proof of concept for each of these different facets has been obtained and we now are armed with a wealth of new technologies and experimental models to develop these innovative therapeutics.

Arguably, the vectors with greatest chance of success in the clinic will combine desirable features, from both cell extrinsic and intrinsic selectivity mechanisms, to generate advanced virotherapies that specifically seek out tumor cells, and only in these unleash their full potency through oncolysis and/or therapeutic transgene expression. The parts of the puzzle of how to build an exquisitely selective virotherapy are therefore emerging. This will surely ultimately guide us toward advanced and effective new gene-based therapies for cancer patients.
